# Reported health, lifestyle and clinical manifestations associated with HIV status in people from rural and urban communities in the Free State Province, South Africa

**DOI:** 10.4102/sajhivmed.v18i1.465

**Published:** 2017-08-28

**Authors:** Michélle Pienaar, Francois C. van Rooyen, Corinna M. Walsh

**Affiliations:** 1 Department of Nutrition and Dietetics, University of the Free State, South Africa; 2 Department of Biostatistics, University of the Free State, South Africa

## Abstract

**Background:**

HIV infection impacts heavily on the infected individual’s overall health status.

**Aim:**

To determine significant health, lifestyle (smoking and alcohol use) and independent clinical manifestations associated with HIV status in rural and urban communities.

**Methods:**

Adults aged between 25 and 64 years completed a questionnaire in a structured interview with each participant. Blood specimens were analysed in an accredited laboratory using standard techniques and controls. Anthropometric measurements were determined using standardised methods.

**Results:**

Of the 567 rural participants, 97 (17.1%) were HIV-infected, and 172 (40.6%) of the 424 urban participants. More than half of HIV-infected rural participants used alcohol and more than 40% smoked. Median body mass index (BMI) of HIV-infected participants was lower than that of uninfected participants. Significantly more HIV-infected participants reported experiencing cough (rural), skin rash (urban), diarrhoea (rural and urban), vomiting (rural), loss of appetite (urban) and involuntary weight loss (rural). Significantly more HIV-uninfected participants reported diabetes mellitus (urban) and high blood pressure (rural and urban). In rural areas, HIV infection was positively associated with losing weight involuntarily (odds ratio 1.86), ever being diagnosed with tuberculosis (TB) (odds ratio 2.50) and being on TB treatment (odds ratio 3.29). In the urban sample, HIV infection was positively associated with having diarrhoea (odds ratio 2.04) and ever being diagnosed with TB (odds ratio 2.49).

**Conclusion:**

Involuntary weight loss and diarrhoea were most likely to predict the presence of HIV. In addition, present or past diagnosis of TB increased the odds of being HIV-infected. Information related to diarrhoea, weight loss and TB is easy to obtain from patients and should prompt healthcare workers to screen for HIV.

## Introduction

Lifestyle factors such as tobacco smoking, use of snuff and alcohol intake impact on quality of life. Cigarette smoking accounts for a large burden of preventable disease in South Africa.^1^ It presents unique health risks in the context of the human immunodeficiency virus (HIV), by increasing receptiveness to HIV or other infections, changing the course of HIV infection itself or altering the risk of smoking-related chronic diseases.^2^ Alcohol consumption is another important risk factor for burden of disease and social destruction worldwide^3,4^ and is discouraged in HIV-infected patients, especially in those on antiretroviral therapy (ART).^5^

In resource-limited countries both infectious and lifestyle diseases contribute to disease burden. Infection with HIV initiates a series of events that ultimately leads to profound immunosuppression caused by functional abnormalities in the immune system, mainly because of severe depletion of CD4+ T cells.^6^

Nutritional alterations are common in HIV infection.^7^ Opportunistic infections affecting the gastrointestinal tract may result in various types of malabsorption,^8^ while advanced immunosuppression from HIV infection can lead to gastrointestinal symptoms such as diarrhoea, nausea, vomiting, dysphagia, weight loss and abdominal pain.^9^ Diarrhoea is a common complaint in patients with HIV infection,^10^ and the severity of symptoms ranges from mild, self-limiting diarrhoea to debilitating disease that can result in malnutrition. Body mass is likely to contribute to the functional impairments seen in patients who are HIV-infected.^11^ Wasting implies unintentional weight loss and loss of lean body mass, and has been strongly associated with an increased risk of disease progression and mortality.^12^ Wasting is associated with chronic diarrhoea, fever or asthenia.^13^

Concurrent HIV and tuberculosis (TB) infection remains a serious challenge. In 2010, 8.8 million people acquired active TB worldwide, of which 1.1 million were living with HIV.^14^ The global burden of TB is increasing, largely because of the spread of HIV,^8^ but these statistics can also be ascribed to other contributing factors such as crowding, poverty, unemployment, malnutrition and poor treatment intervention.^8^

Lifestyle diseases are the leading cause of death globally, killing more people each year than all other causes combined.^14^ In the general population, chronic lifestyle diseases share similar risk factors, including tobacco smoking, diabetes mellitus, hypertension (often associated with increasing age^15^), obesity, hyperlipidaemia and physical inactivity.^16^ The presence of comorbidities such as heart disease, diabetes mellitus, hepatitis and opportunistic infections may also complicate the profile of the HIV-infected patient on ART.^17^

This study formed part of the Assuring Health for All in the Free State (AHA-FS) study, which aimed to determine how living in rural and urban communities can influence lifestyle and health. Despite the large body of evidence related to clinical and anthropometric manifestations of HIV, epidemiological data on these manifestations in the Free State Province are limited. The clinical relevance of identifying variables that are likely to predict HIV lies in improved screening, diagnosis and care of the large numbers of patients that visit primary healthcare facilities. The aim of this sub-study was thus to investigate health (history of disease, medication, anthropometric symptoms experienced) and lifestyle (smoking and alcohol use) of HIV-infected and HIV-uninfected, rural and urban respondents and to determine significant independent clinical manifestations associated with HIV status.

## Methods

The rural study was performed in three Free State towns, namely, Trompsburg, Philippolis and Springfontein, and the urban study in Mangaung.

### Study design, target population and sampling

A cross-sectional study was undertaken. In rural areas, all households were eligible to participate. Before data were collected, induction meetings for community members and other role-players were arranged in each community. The role-players included clinic staff, church leaders, community leaders and any members of the community who were interested in learning more about the project or had questions that they wanted to ask. These role-players informed community members that were not present at induction meetings that all adults that met the inclusion criteria were welcome to participate. On days of data collection, adults that arrived at the research venue were included in the rural sample.

In urban Mangaung, the number of plots in the Mangaung University Community Partnerships Programme (MUCPP) service area was counted on a municipal map and included Buffer, Freedom Square, Kagisanong, Chris Hani, Namibia and Turflaagte. An estimate was made of additional squatter households in open areas. A stratified proportional cluster sample was selected, stratified by area and formal plot or squatter households in open areas. Using randomly selected X and Y coordinates, 100 starting points were selected in this way. From each point, five adjacent starting households were approached to participate in the study. Every adult member of households in these black and mixed-ethnic communities, who gave informed consent and was between 25 and 64 years of age, was eligible to participate.

### Pilot study

Prior to the main survey, a pilot study was undertaken with five individuals in each area, similar to the target group, in order to determine whether questions included in the questionnaire could be easily understood and to estimate the amount of time needed to complete the questionnaires. The questionnaire and all anthropometric measurements were piloted. Minor changes (mostly technical editing) were made to questionnaire after the pilot study.

### Variables and operational definitions

For the purpose of this study, health referred to medical history, medication used, hospitalisation, anthropometry and symptoms experienced. Lifestyle referred to tobacco and alcohol consumption patterns. Among others, laboratory investigations included HIV status.

Anthropometric variables included height, weight and waist circumference (WC). Adults were categorised as underweight (body mass index [BMI] less than 18.5 kg/m^2^); normal weight (BMI 18.5 kg/m^2^ or over, but less than 25 kg/m^2^); overweight (BMI 25 kg/m^2^ or over, but less than 30 kg/m^2^); or obese (BMI 30 kg/m^2^ or over).^18^ A WC equal to or larger than 94 cm for men and 80 cm for women was considered as a high risk for lifestyle-related diseases.^18^

### Methods and techniques

A health questionnaire, adapted from the one developed for the Prospective Urban Rural Epidemiology (PURE) study,^19^ was completed for all adults in each household. Information was collected in an interview with each adult by trained final-year dietetics students, using a structured questionnaire. To assure validity, all questions in the health questionnaire were related to the objectives of the study and were based on health-related issues associated with HIV in relevant literature. Random samples of 10% of the rural and urban participants were interviewed a second time by the researchers to determine reliability of questions asked in the health questionnaire within a month of the initial survey. Where the percentage of answers to questions differed with more than 20%, the question was considered unreliable. No questions were found to be unreliable in the health questionnaire. All blood specimens were analysed in an accredited laboratory using standard techniques and controls. Anthropometric measurements were taken with respondents wearing an examination gown, without shoes. Anthropometric measurements were performed by trained dietetics students, using standardised methods.^20^

### Data collection

Data collection took place at different research venues, including the community hall in the rural area or at the MUCPP nutrition centre in the urban area. On days of data collection, identity documents were screened in order to make sure that participants met the inclusion criteria with regard to age. The research venues included stations for the collection of blood samples; a food station; medical examination; anthropometric measurements (participants arrived in a fasting state for the collection of blood samples). Thereafter, questionnaires related to the following were completed: sociodemography; household food security; dietary intake; physical activity; and self-reported health.

### Statistical analysis

All analyses were performed by the Department of Biostatistics, UFS. Descriptive statistics, including frequencies and percentages for categorical data, and means and standard deviations (SDs) for symmetrical numerical variables, or medians and percentiles for skew numerical variables, were calculated. Differences between HIV-infected and HIV-uninfected groups were assessed by *p*-values (*t*-tests [for symmetrical numerical variables], Mann–Whitney tests [for skew numerical variables], chi-squared tests [for categorical variables] or Fischer’s extract test [for categorical variables with sparse data]) or 95% confidence intervals (CIs) for median, mean or percentage differences.

In addition to descriptive comparisons between HIV-infected and HIV-uninfected participants, logistic regression was applied to identify significant clinical manifestations associated with HIV. For each of the variables, a univariate analysis was applied to identify variables that could be included in the rural and urban model (*p* < 0.15). In the rural sample, the following health variables were included in the model: history of alcohol use, loose stools or diarrhoea for at least three days in last six months, involuntary weight loss > 3 kg in last six months, ever diagnosed with TB, family member diagnosed with TB and TB treatment.

In the urban sample, the health variables that were included in the model were: loose stools or diarrhoea for at least three days in last six months, loss of appetite in last six months, involuntary weight loss > 3 kg in last six months, ever diagnosed with TB and TB treatment.

Following this, logistic regression with forward selection (*p* < 0.05) was applied to select significant independent factors associated with HIV status in the rural and urban samples.

## Ethical consideration

The study was approved by the Ethics Committee of the Faculty of Health Sciences at the UFS (ETOVS 21/07), the Free State Department of Health (DoH) and local municipalities. The researchers obtained written consent from all participants in their language of choice.

## Results

Of the 570 rural participants, 567 had HIV results. Of these, 97 (17.1%) were HIV-infected. Of the 426 urban participants, 424 had HIV results, of which 172 (40.6%) were HIV-infected. Twenty-five per cent (*n* = 43) of the total number of HIV-infected urban respondents reported using ART, compared to only four (4.1%) HIV-infected respondents in rural areas.

### Smoking and snuffing

Results related to smoking and use of snuff are shown in Table 1. In both areas, more or less the same percentage of HIV-infected participants smoked or used snuff compared to HIV-uninfected participants. A fairly large percentage of rural participants smoked (40.9% HIV-infected and 39.6% HIV-uninfected participants), while one in four urban participants used snuff (25% of both HIV-infected and HIV-uninfected participants).

**TABLE 1:** History of smoking and use of snuff.

Variable	Sample	Rural					Urban				
		HIV-positive		HIV-negative		*p*†	HIV-positive		HIV-negative		*p*‡
		*n*	%	*n*	%		*n*	%	*n*	%	
**History of smoking**	RHP 88, RHN 452; UHP 164, UHN 244	-	-	-	-	-	-	-	-	-	-
Never smoked	-	35	39.8	190	42.0	-	113	68.9	162	66.4	-
Currently smoke	-	36	40.9	179	39.6	0.81	39	23.8	52	21.3	0.55
Formerly smoked	-	17	19.3	83	18.4	-	12	7.3	30	12.3	-
**History of use of snuff**	RHP 87, RHN 449; UHP 164, UHN 244	-	-	-	-	-	-	-	-	-	-
Never used snuff	-	68	78.2	346	77.1	-	111	67.7	170	69.7	-
Currently use snuff	-	15	17.2	79	17.6	0.93	41	25.0	62	25.4	0.92
Formerly used snuff	-	4	4.6	24	5.4	-	12	7.3	12	4.9	-

† , *p*-value for difference between HIV-positive and HIV-negative rural participants using chi-squared or Fischer’s exact test, as appropriate;

‡ , *p*-value for difference between HIV-positive and HIV-negative urban participants using chi-squared or Fischer’s exact test, as appropriate.

RHP, rural, HIV-positive; RHN, rural, HIV-negative; UHP, urban, HIV-positive; UHN, urban, HIV-negative.

### Alcohol consumption

Table 2 shows categories of alcohol consumption of HIV-infected and HIV-uninfected rural and urban respondents. More HIV-infected participants used alcohol than their uninfected counterparts (54.6% vs. 47.6% [rural] and 42.3% vs. 36.5% [urban]), although the difference was not significant. Beer was the most frequently consumed alcoholic beverage in all groups, but more rural respondents consumed homemade beer, ranging from 47.8% of HIV-infected participants to 57.9% of HIV-uninfected participants. More HIV-infected rural respondents (84.2%) felt tired on Mondays after heavy drinking than uninfected rural participants (61.8%), but the difference was not statistically significant (*p* = 0.07).

**TABLE 2:** Alcohol consumption.

Variable	Sample	Rural					Urban				
		HIV-positive		HIV-negative		*p*†	HIV-positive		HIV-negative		*p*‡
		*n*	%	*n*	%		*n*	%	*n*	%	
**History of alcohol use**	RHP 88, RHN 450; UHP†† 163, UHN 244										
Never used alcohol	-	13	14.8	100	22.2	-	68	41.7	118	48.4	-
Currently use alcohol	-	48	54.6	214	47.6	0.23	69	42.3	89	36.5	0.22
Formerly used alcohol	-	27	30.7	136	30.2	-	26	16.0	37	15.2	-
**If currently, what form**											
Spirits	RHP 47, RHN 204; UHP 66, UHN 84	10	21.3	34	16.7	-	7	10.6	5	6.0	-
Wine	RHP 47, RHN 206; UHP 65, UHN 83	8	17.0	41	19.9	-	8	12.3	10	12.1	-
Beer, cider	RHP 46, RHN 205; UHP 66, UHN 83	31	67.4	114	55.6	-	47	71.2	54	65.1	-
Homemade beer	RHP 45, RHN 202; UHP 65 UHN 83	22	47.8	117	57.9	-	13	20.0	24	28.9	-
**At least once a month consume > 5 drinks/day**	RHP 48, RHN 206; UHP 66, UHN 88	21	43.8	88	42.7	0.89	26	39.4	34	41.0	0.84
**Feel tired on Monday after heavy alcohol consumption (> 5 drinks/day)**	RHP 19, RHN 68; UHP 22, UHN 27	16	84.2	42	61.8	0.07	15	68.2	22	81.5	0.28

† , *p*-value for difference between HIV-positive and HIV-negative rural participants using chi-squared or Fisher’s exact test, as appropriate;

‡ , *p*-value for difference between HIV-positive and HIV-negative urban participants using chi-squared or Fisher’s exact test, as appropriate;

RHP, rural, HIV-positive; RHN, rural, HIV-negative; UHP, urban, HIV-positive; UHN, urban, HIV-negative.

### Reported symptoms, diagnoses and medication use

Reported symptoms and medication use of HIV-infected and HIV-uninfected rural and urban participants are described in Table 3. HIV-infected participants in both areas reported that they had experienced significantly more loose stools and diarrhoea compared to HIV-uninfected participants (rural = 38.2% vs. 27.7%, *p* = 0.04; urban = 32.9% vs. 19.8%, *p* = 0.002). Rural HIV-infected respondents experienced significantly more vomiting than HIV-uninfected counterparts (32.6% vs. 21.8%, *p* = 0.02) and involuntary weight loss of more than 3 kg (64.0% vs. 50.3%, *p* = 0.01). Significantly more rural HIV-infected respondents had experienced chest pain or tightness (60.7% vs. 46.8%, *p* = 0.02) and cough for at least two weeks (53.9% vs. 40.8%, *p* = 0.02) compared to HIV-uninfected rural participants. On the other hand, significantly more HIV-uninfected rural participants reported joint pain (70.1% vs. 55.7%, *p* = 0.008), as well as high blood pressure (66.1% vs. 44.9%, *p* = 0.002) compared to their HIV-infected counterparts.

**TABLE 3:** Reported symptoms, diagnoses and medication use.

Variable	Sample	Rural					Urban				
		HIV-positive		HIV-negative		*p*†	HIV-positive		HIV-negative		*p*‡
		*n*	%	*n*	%		*n*	%	*n*	%	
**Experienced during last 6 months**											
Loose stools, diarrhoea for at least 3 days	RHP 89, RHN 451, UHP 164, UHN 243	34	38.2	125	27.7	0.04*	54	32.9	48	19.8	0.002*
Vomiting	RHP 89, RHN 450, UHP 164, UHN 244	29	32.6	98	21.8	0.02*	41	25.0	47	19.3	0.16
Loss of appetite	RHP 89, RHN 451, UHP 164, UHN 244	41	46.1	184	40.8	0.35	95	57.9	111	45.5	0.01*
Involuntary weight loss > 3 kg	RHP 89, RHN 451, UHN 164, UHN 244	57	64.0	227	50.3	0.01*	78	47.6	92	37.7	0.05
Chest pain or tightness with usual activity	RHP 89, RHN 451; UHP 164, UHN 243	54	60.7	211	46.8	0.02*	97	59.2	137	56.4	0.57
Cough for at least 2 weeks	RHP 89, RHN 451, UHP 164, UHN 244	48	53.9	184	40.8	0.02*	74	45.1	96	39.3	0.24
Wheezing or whistling in chest	RHP 89, RHN 451, UHP 164, UHN 244	41	46.1	195	43.2	0.62	62	37.8	86	35.3	0.59
Sexually transmitted diseases	RHP 89, RHN 452, UHP 164, UHN 244	12	13.5	32	7.1	0.04*	56	34.2	13	5.3	0.0001*
Blood in urine	RHP 89, RHN 452, UHP 164, UHN 244	11	12.4	32	7.1	0.09	11	6.7	18	7.4	0.79
Skin rash	RHP 89, RHN 451, UHP 164, UHN 244	26	29.2	115	25.5	0.46	62	37.8	60	24.6	0.004*
Breathlessness with usual activity	RHP 89, RHN 452, UHP 164, UHN 244	53	59.6	223	49.3	0.07	81	49.4	129	52.9	0.49
Swelling of feet	RHP 89, RHN 452, UHP 164, UHN 244	32	36.0	177	39.2	0.57	67	40.9	124	50.8	0.04*
Joint pain	RHP 88, RHN 451, UHP 164, UHN 244	49	55.7	316	70.1	0.008*	96	58.5	163	66.8	0.09
**Diagnosed with the following**											
Liver disease, hepatitis, jaundice	RHP 89, RHN 452, UHP 164, UHN 244	4	4.5	12	2.7	0.31	12	7.3	7	2.9	0.03*
Lung disease, e.g. emphysema or asthma	RHP 89, RHN 451, UHP 164, UHN 244	13	14.6	58	12.9	0.65	14	8.5	18	7.4	0.66
TB	RHP 88, RHN 452, UHP 164, UHN 244	24	27.3	46	10.2	0.0001*	40	24.4	26	10.7	0.0002*
Diabetes mellitus	RHP 89, RHN 450, UHP 164, UHN 244	5	5.6	55	12.2	0.07	7	4.3	25	10.3	0.02*
High blood pressure	RHP 89, RHN 451, UHP 164, UHN 244	40	44.9	298	66.1	0.002*	58	35.4	139	57.0	0.0001*
Stroke	RHP 89, RHN 452, UHP 164, UHN 244	8	9.0	27	6.0	0.29	9	5.5	11	4.5	0.65
Heart disease, angina, heart attack	RHP 89, RHN 448, UHP 164, UHN 244	14	15.7	69	15.4	0.93	27	16.5	43	17.6	0.76
Heart failure	RHP 89, RHN 452, UHP 164, UHN 244	1	1.1	5	1.1	1.0	8	4.9	11	4.5	0.86
Cancer	RHP 89, RHN 452, UHP 164, UHN 244	0	0.0	6	1.3	0.59	3	1.8	6	2.5	0.74
Epilepsy	RHP 89, RHN 451, UHP 164, UHN 244	4	4.5	22	4.9	1.0	11	6.7	14	5.7	0.68
Allergy	RHP 89, RHN 451, UHP 164, UHN 244	12	13.5	68	15.1	0.69	28	17.1	45	18.4	0.72
**Medication**											
Taking medication regularly	RHP 88, RHN 448, UHP 164, UHN 244	62	70.5	347	77.5	0.16	90	54.9	132	54.1	0.87
Type of medication	RHP 62, RHN 347, UHP 90, UHN 132	-	-	-	-	-	-	-	-	-	-
ART	-	4	6.5	0	0.0	0.0001*	43	47.8	0	0.0	-
TB treatment	-	10	16.1	8	2.3	0.0001*	10	11.1	4	3.0	0.01*
Diabetes (oral)	-	2	3.2	42	12.1	0.02*	0	0.0	13	9.9	0.0027*
Hypertension	-	28	45.2	252	72.6	0.0001*	22	24.4	90	68.2	0.0001*
Other	-	54	87.1	314	90.5	-	46	51.1	49	37.1	-
**Hospitalised during past 12 months**	RHP 88; RHN 451, UHP 164, UHN 244	26	29.6	104	23.1	-	46	28.1	65	26.6	-

† , *p*-value for difference between HIV-positive and HIV-negative rural participants using chi-squared or Fisher’s exact test, as appropriate;

‡ , *p*-value for difference between HIV-positive and HIV-negative urban participants using chi-squared or Fisher’s exact test, as appropriate;

TB, tuberculosis; RHP, rural, HIV-positive; RHN, rural, HIV-negative; UHP, urban, HIV-positive; UHN, urban, HIV-negative.

* , Statistically significant difference.

In urban areas, significantly more HIV-infected urban participants reported loose stools or diarrhoea for at least three days (32.9% vs. 19.8%, *p* = 0.002), loss of appetite (57.9% vs. 45.5%, *p* = 0.01), skin rash (37.8% vs. 24.6%, *p* = 0.004), as well as liver diseases, hepatitis or jaundice (7.3% vs. 2.9%, *p* = 0.03), compared to HIV-uninfected respondents. In contrast, more HIV-uninfected participants reported swelling of feet (50.8% vs. 40.9%, *p* = 0.04), diabetes mellitus (10.3% vs. 4.3%, *p* = 0.02) and hypertension (57.0% vs. 35.4%, *p* = 0.0001) than their HIV-infected counterparts.

HIV-infected participants in both groups were significantly more likely to have been diagnosed with TB (27.3% vs. 10.2% in rural areas [*p* = 0.0001] and 24.4% vs. 10.7% in urban areas [*p* = 0.0002]). In both areas, significantly more HIV-uninfected participants compared to HIV-infected participants were on medication to treat diabetes (12.1% vs. 3.2%, *p* = 0.02) and hypertension: 72.6% vs. 45.2% in rural areas (*p* = 0.0001) and 68.2% vs. 24.4% in urban areas (*p* = 0.0001). On the other hand, significantly more HIV-infected participants took TB medication than their uninfected counterparts: 16.1% vs. 2.3% in rural areas (*p* = 0.0001) and 11.1% vs. 3.0% in urban areas (*p* = 0.01).

### Anthropometric information

In rural areas, the median BMI of HIV-uninfected men fell into the normal weight category at 21.0 kg/m^2^ compared to 18.7 kg/m^2^ of the HIV-infected men, indicating a median difference of 2.3 kg/m^2^, which was statistically significant (*p* = 0.02) (Table 4). The median BMI of HIV-infected rural women fell within the normal range at 23.1 kg/m^2^, while the median BMI of HIV-uninfected rural women fell in the overweight category at 27.7 kg/m^2^, with a significant median difference of 4.6 kg/m^2^ (*p* = 0.009).

**TABLE 4:** Body mass index of HIV-infected and HIV-uninfected participants in rural and urban communities.

Community	Gender	HIV-positive						HIV-negative						*p*†
		*n*	Median	Mean	SD	Min	Max	*n*	Median	Mean	SD	Min	Max	
Rural	Male	25	18.7	19.6	4.5	15.5	33.6	85	21.0	21.84	5.27	11.8	41.4	0.02*
Urban		36	19.4	19.8	2.58	14.5	25.3	61	20.9	22.64	6.6	14.7	49.9	0.07
Rural	Female	63	23.1	24.9	6.8	13.7	42.5	359	27.7	28.8	8.9	11.9	53.6	0.009*
Urban		131	25.0	26.0	7.6	13.3	55.7	185	31.8	32.4	8.8	14.5	55.1	0.001*

† , for median difference.

* , statistically significant at *p* < 0.05.

SD, standard deviation.

The median BMI of HIV-infected and uninfected urban men fell within the normal weight category at 19.4 kg/m^2^ and 20.9 kg/m², respectively, a difference which was not statistically significant (*p* = 0.07). The median BMI of HIV-infected and HIV-uninfected urban women fell within the overweight and obese category at 25.0 kg/m^2^ and 31.8 kg/m^2^, respectively, with a significant median difference of 6.8 kg/m^2^ (*p* = 0.001).

A larger percentage of HIV-infected women had a WC below 80 cm compared to HIV-uninfected women, a difference that was statistically significant (*p* = 0.0003 in rural and *p* = 0.0001 in urban areas). Significantly more HIV-uninfected women tended to have a WC in the ‘high risk’ category (WC of more than 88 cm) compared to HIV-infected women (*p* = 0.0003 in rural areas and *p* = 0.0001 in urban areas).

### Reported clinical manifestations associated with HIV status in rural participants

Logistic regression was applied to identify significant clinical manifestations associated with HIV in the rural (Table 5) and urban (Table 6) samples.

**TABLE 5:** Reported clinical manifestations associated with HIV status (rural).

Variable	Yes vs. no	Odds ratio	95% CI	*p*
Age	–	0.02	0.89; 0.95	< 0.0001
Involuntary weight loss > 3 kg in last 6 months	Yes vs. no	1.86	1.08; 3.20	0.0255
Ever diagnosed with TB	Yes vs. no	2.50	1.18; 5.23	0.0163
TB treatment	Yes vs. no	3.29	1.00; 10.80	0.0498

TB, Tuberculosis; CI, confidence interval; vs, versus.

**TABLE 6:** Reported clinical manifestations associated with HIV status (urban).

Variable	Yes vs. no	Odds ratio	95% CI	*p*
Age	–	0.93	0.91; 0.95	< 0.0001
Loose stools or diarrhoea for at least 3 days in last 6 months	Yes vs. no	2.04	1.23; 3.41	0.0061
Ever diagnosed with TB	Yes vs. no	2.49	1.37; 4.53	0. 0028

TB, Tuberculosis; CI, confidence interval; vs, versus.

In the rural sample, for every year that age increased, the odds of having HIV decreased by 8%. HIV infection was positively associated with losing weight involuntarily (> 3 kg in the past six months; odds ratio 1.86), ever being diagnosed with TB (odds ratio 2.50) and being on TB treatment (odds ratio 3.29).

In the urban sample, for every year that age increased, the odds of having HIV decreased by 7%. HIV infection was positively associated with having diarrhoea for at least three days in the past six months (odds ratio 2.04) and ever being diagnosed with TB (odds ratio 2.49).

## Discussion

The high prevalence of HIV in urban Mangaung has been reported previously.^21^ In sub-Saharan Africa, the HIV epidemic was traditionally concentrated in urban areas, where significantly higher HIV prevalence rates have been recorded than in rural areas.^22^ Possible reasons may include a correlation between HIV and the level of urbanisation in South Africa, a higher percentage of younger people living in urban areas, poor living conditions (including a lack of food security) and gender relations.^22^ In addition to HIV-related burden of disease, the high prevalence of obesity and overweight has reached epidemic proportions in South African communities.^23^

A fairly large percentage of participants in the current study smoked and used snuff. Lifestyle factors such as tobacco smoking, use of snuff and alcohol intake impact on quality of life of both the general population, as well as those with HIV. More HIV-infected participants tended to use alcohol than their uninfected counterparts, with beer being the most frequently consumed alcoholic beverage in all groups. Alcohol may be a mediating factor in risky sexual behaviour and therefore impacts on the transmission of HIV.^24^ In addition, heavy drinking may lead to people on treatment not taking their medication properly.^25^

In the early period, infectious diseases such as HIV and lifestyle diseases were perceived to be largely different.^26^ However, more and more HIV-infected persons are using ART, resulting in them living longer and developing chronic conditions similar to the rest of the population.

Significantly more HIV-infected participants reported experiencing loose stools and diarrhoea, as well as involuntary weight loss, compared to HIV-uninfected participants, and were therefore at higher risk of developing malnutrition. Diarrhoea affects 40–80% of HIV-infected adults who do not receive ART.^27^ This was confirmed in this study where the majority of HIV-infected participants were not using ART (only 25% of all HIV-infected urban participants and 4.1% of rural HIV-infected participants reported using ART). At the time that the study was undertaken, ART was only available at one clinic in the rural Southern Free State, namely Jagersfontein, possibly explaining why such a low percentage of HIV-infected participants in the rural areas were accessing ART. Some common nutritional challenges in HIV-infected individuals include inadequate oral food intake; increased nutrient needs; swallowing difficulty; altered gastrointestinal function; food–medication interactions; involuntary weight loss and impaired ability to prepare foods and meals.^12^ Food insecurity (also observed in this study) is also an important aspect of HIV-associated weight loss and wasting. It is generally complicated further by the fact that individuals who are ill are not able to go to work to earn enough money to buy food.^8^

In the current study, logistic regression indicated that HIV infection was positively associated with losing weight involuntarily (more than 3 kg in the past six months) (rural) and having diarrhoea for at least three days in the past six months (urban). Previous research has shown significant weight loss and a high prevalence of underweight among HIV-infected adults.^28^ Up to 40% of patients with HIV infection report at least one episode of diarrhoea in a given month, and approximately one quarter of patients experience chronic diarrhoea at some point.^10^

South Africa has a high TB–HIV co-infection rate of 73%, yet only 46% of TB patients are tested for HIV.^29^ Results from the logistic regression in this study confirmed that HIV infection was positively associated with a history of TB diagnosis (rural and urban) as well as TB treatment (rural). This finding is similar to a retrospective analysis carried out in Cape Town by Osman et al.,^30^ reporting that HIV-infected and HIV-unknown patients with TB have an increased risk of death during TB treatment.

In the current study a larger percentage of HIV-uninfected participants reported having diabetes mellitus and hypertension. This could probably be ascribed to the lower median BMI and WC of HIV-infected participants. A similar trend has also been reported among HIV-infected black women in Mangaung.^31^

Overnutrition is prevalent among the general population of adult South Africans, particularly women.^32^ Although the prevalence of overweight and obesity was lower in HIV-infected participants in the current study, a large proportion of both HIV-infected and HIV-uninfected women were still overweight and obese, while men were more likely to have a normal or low BMI. Epidemiological studies have shown gender differences in the prevalence of obesity in African nations, with levels of overweight considerably higher in females than males.^32,33,34^ The younger mean age of HIV-infected participants should also be kept in mind, because weight gain is often the result of ageing.

The results of the current study confirm that the clinical and anthropometric manifestations of HIV infection reported in the literature are also evident in HIV-infected persons from the Free State. The high prevalence of overweight (among women) associated with symptoms such as weight loss and diarrhoea that were identified in the participants in the current study, can complicate the management of HIV-infected patients in the primary healthcare setting. Information about unintentional weight loss, diarrhoea and a history of TB can prompt healthcare professionals to screen for HIV, even in women who are not underweight.

We acknowledge that there could have been a certain degree of bias because older and unemployed individuals were more likely to participate. It is also possible that because of limited health services, ill persons might have been more likely to participate in the study where medical examinations were conducted, especially in rural areas. Furthermore, the younger age and lower BMI of HIV-infected participants complicate a comparison of clinical manifestations of HIV-infected participants to those of HIV-uninfected participants. Because of these reasons, the authors are aware that the study group is probably not representative of the general population.

## Conclusion

Involuntary weight loss (rural) and diarrhoea (urban) were most likely to predict HIV infection. In both samples, a history of TB (rural and urban) or TB treatment (rural) was positively associated with HIV infection. In addition, median BMI and WC of HIV-infected respondents were significantly lower than in HIV-uninfected respondents (although many HIV-infected women were still in the overweight category). The HIV-uninfected group consequently had a higher occurrence of lifestyle diseases, such as diabetes mellitus and hypertension.

The results of the current study confirm the higher prevalence of opportunistic infections and the associated symptoms (such as diarrhoea and weight loss) in HIV-infected persons in this sample. Information related to diarrhoea, weight loss and past or present TB is easy to obtain from patients and should prompt healthcare workers to screen patients for HIV and to implement relevant interventions.
